# Claw Lesions Causing Clinical Lameness in Lactating Holstein Frisian Crossbred Cows

**DOI:** 10.1155/2014/764689

**Published:** 2014-07-14

**Authors:** Umar Nazir Zahid, Swaran Singh Randhawa, Syed Ashaq Hussain, Sarnarinder Singh Randhawa, Vishal Mahajan, Kirti Dua

**Affiliations:** ^1^Department of Animal Husbandry, Jammu and Kashmir 190019, India; ^2^Department of Veterinary Medicine, Guru Angad Dev Veterinary and Animal Sciences University, Ludhiana, Punjab 141004, India; ^3^Guru Angad Dev Veterinary and Animal Sciences University, Ludhiana, Punjab 141004, India; ^4^Animal Disease Research Centre, Guru Angad Dev Veterinary and Animal Sciences University, Ludhiana, Punjab 141004, India

## Abstract

The objective of this study was to identify claw lesions causing clinical lameness in lactating Holstein Frisian (HF) crossbred cows in dairy cattle. Seventy dairy farmers were interviewed at the monthly meetings of Progressive Dairy Farmers Association of Ludhiana, Punjab, India. Ten dairy farms were randomly selected as per probability proportional to size and a total of 450 lactating HF crossbred cows were taken into the study. All the lactating cows were scored for locomotion and rear leg view index. Trimming was done in all the clinically lame animals (animals with locomotion scores 2 and 3) and equal number of animals selected randomly from those with locomotion scores 0 and 1. Various claw lesions were evaluated in both the groups. There was a significant relationship between locomotion score and rear leg view index to identify lameness. Sole ulcers and white line fissures were the lesions responsible for clinical lameness. Other lesions did not cause clinical lameness but increased the asymmetry in lactating HF crossbred cows. Both locomotion score and rear leg view index could be reliably used to identify clinical lameness in lactating cattle.

## 1. Introduction

Lameness is one of the greatest economic concerns of present day dairy industry. The major ill effects of lameness include pain, distress, loss in production, a negative impact on reproductive performance, and an increased risk of culling [1]. It is reported that 60% of herd may become lame at least once a year [2] and about 90–99% of lameness incidents occur due to claw lesions [3, 4]. Clinical lameness is comparatively more concerned because of high rate of culling [5] and marked reduction in milk yield [6].

The mere presence of a lesion is not associated with clinical lameness [7] but it also depends on severity of foot lesion. Only a few studies have investigated the relationship between locomotion score and the type of foot lesion present [8–10]. Sole ulcer, double sole, interdigital purulent inflammation, and severe stages of digital dermatitis have been associated with clinical lameness [7, 11]. Keeping in view the increasing population of HF crossbred cows in Punjab, India, this study seemed to be necessary. The present study was designed to identify the foot lesions responsible for clinical lameness in lactating HF crossbred cattle. Another objective was to evaluate the reliability of locomotion score and rear leg view index for identifying clinical lameness.

## 2. Materials and Methods

### 2.1. Selection of Animals

Seventy farmers were interviewed at the monthly meetings of Progressive Dairy Farmers Association of Ludhiana, Punjab, India. The farmers were questioned about general management, housing system, claw trimming routines, milk production, nutrition, number of lame animals, presence of lameness chute, and knowledge about lameness and its ill effects. Among these seventy enrolled farmers, ten farms were selected randomly as per probability proportional to size sampling [12]. A total of 450 lactating HF cross bred cows were included in the study. All these animals were kept in loose housing system with provision of both soft and concrete flooring. The average milk yield of these animals was 3000–4000 litres/lactation. Majority of the animals were fed maize silage along with concentrate feed @ 400 gram for every kilogram of milk production.

### 2.2. Locomotion Score and Rear Leg View Index (RLVI)

All the ten farms were visited after the morning milking. Locomotion score and rear leg view index of all the 450 animals were evaluated independently by two observers, working together. For locomotion score, the cows were allowed to walk on a flat surface for up to 30 meters. Locomotion score of each animal was assessed on a five-point scale as described by Wells et al. [13]. RLVI of each animal was recorded as normal/score 0 (when there was no inward knuckling of hocks and hocks were straight) and cow hock/score 1 (when there was inward knuckling of hocks), when observed from rear side [14].

### 2.3. Hoof Examination

Hoof trimming was done as per standard procedure [14] and different foot lesions were observed in all the lame animals (locomotion scores 2 and 3) and equal number of animals selected randomly from those with locomotion scores 0 and 1 [12]. Each animal was properly restrained in trimming chute and then each foot was examined for any lesion before and after paring a layer of approximately 1 mm of horn from the weight bearing surface. Heel erosions, sole haemorrhages, sole avulsions, white line haemorrhages, and white line fissures were scored for severity on hoof maps, as per Randhawa et al. [15]. The rest of the foot lesions, namely, sole ulcers, underrun soles, double soles, overgrown hooves, overgrown soles, and interdigital hyperplasia, were categorized as 1 (when present) and 0 (when absent) [14].

### 2.4. Statistical Analysis

In order to identify the lesions responsible for lameness, regression analysis was carried out using statistical package for social sciences (SPSS for windows version 11-0-1; SPSS Inc, Chicago, Illinois). The correlation between different lesions within cow was investigated and correlated at *P* ≤ 0.05 and *P* ≤ 0.01. The relationship between locomotion score and rear leg view was analyzed by Chi square analysis. The reliability of locomotion scores assigned by two observers was analyzed by calculating Spearman's rank correlation coefficient.

## 3. Results

**Figure 1:**
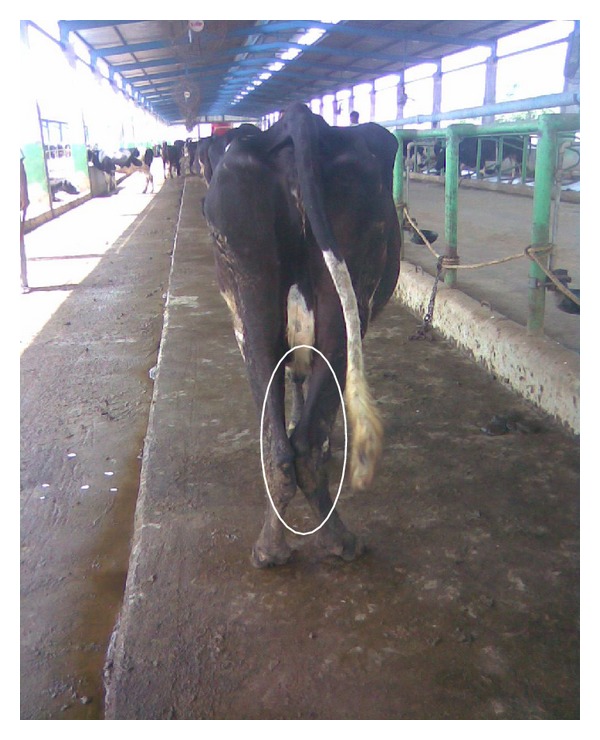
Cow hock: rear leg view index.

**Figure 2:**
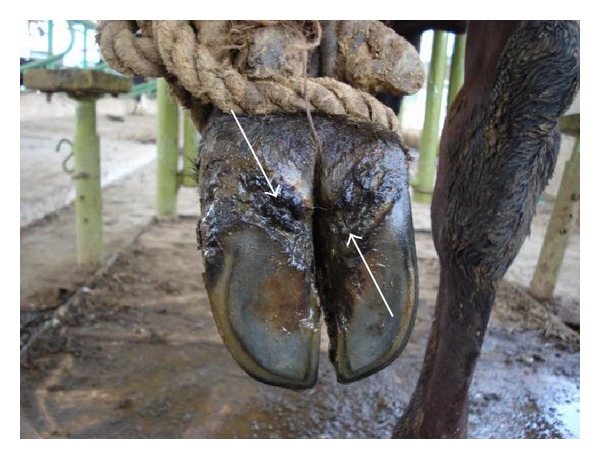
Heel erosion.

**Figure 3:**
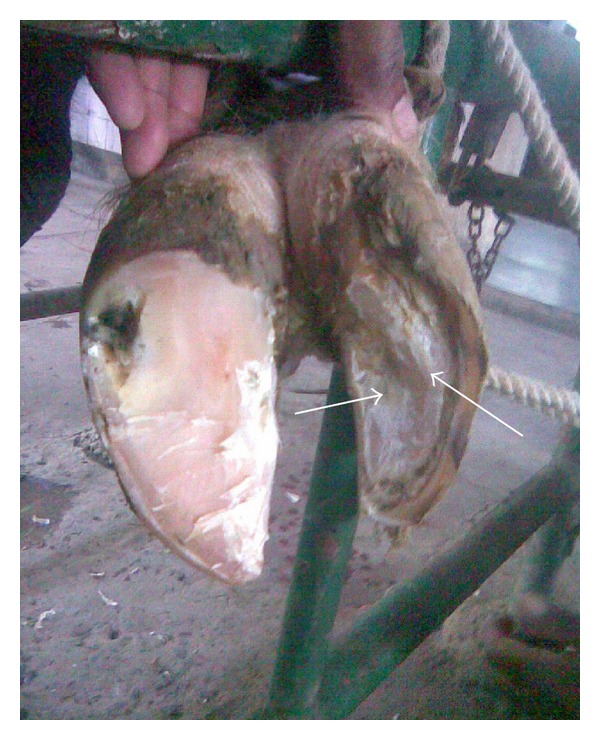
Sole avulsion.

**Figure 4:**
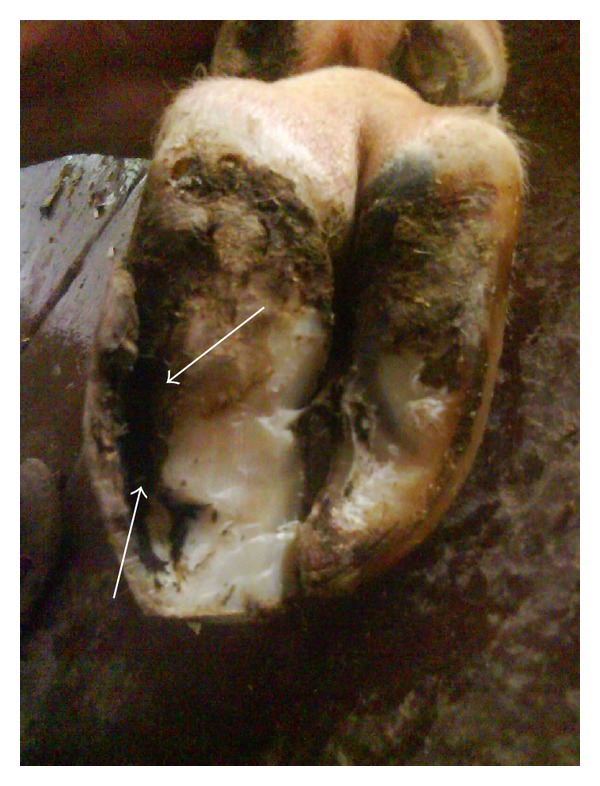
White line fissure.

**Figure 5:**
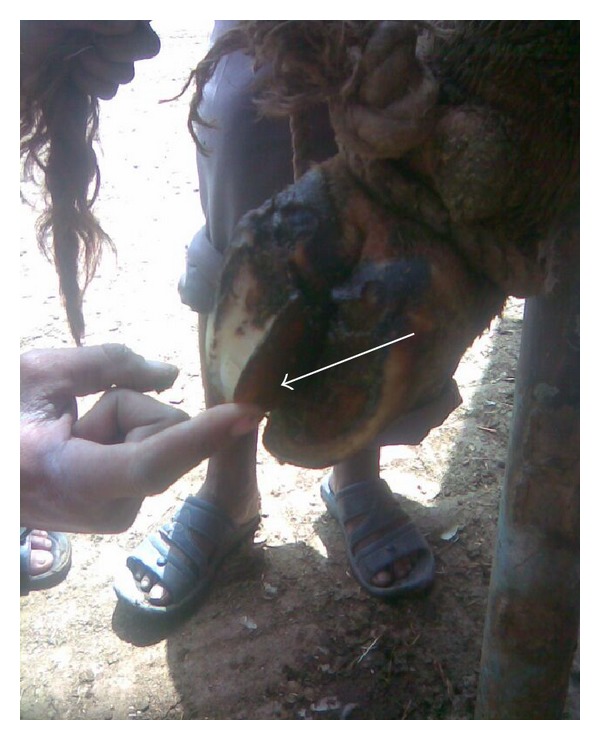
Underrun sole.

**Figure 6:**
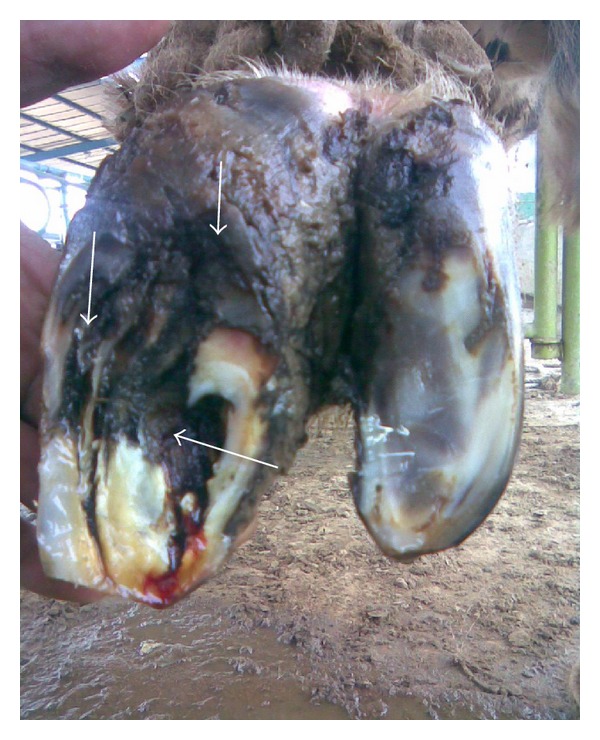
Heel erosion, white line fissure, and underrun sole.

**Figure 7:**
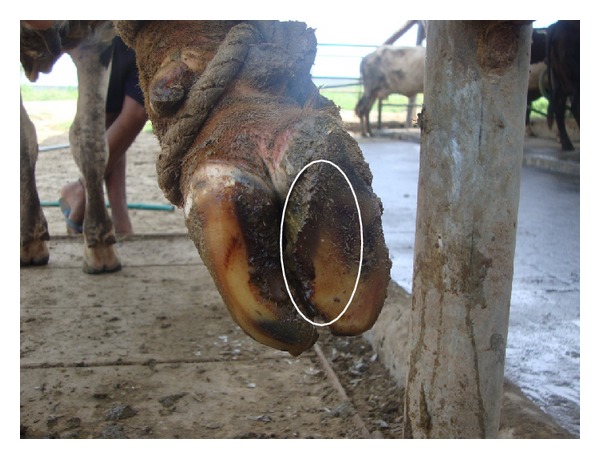
Overgrown sole.

**Figure 8:**
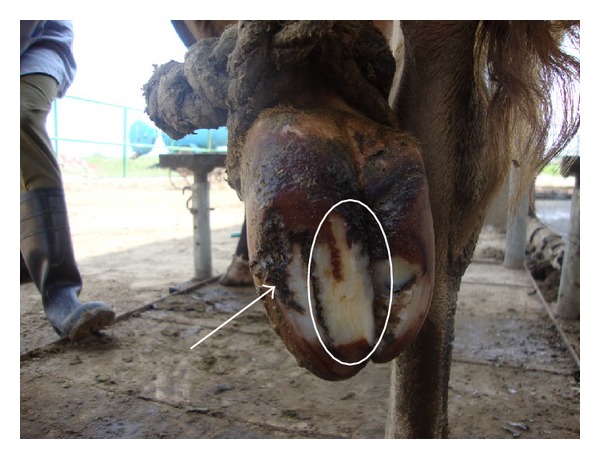
Overgrown sole and white line fissure.

**Figure 9:**
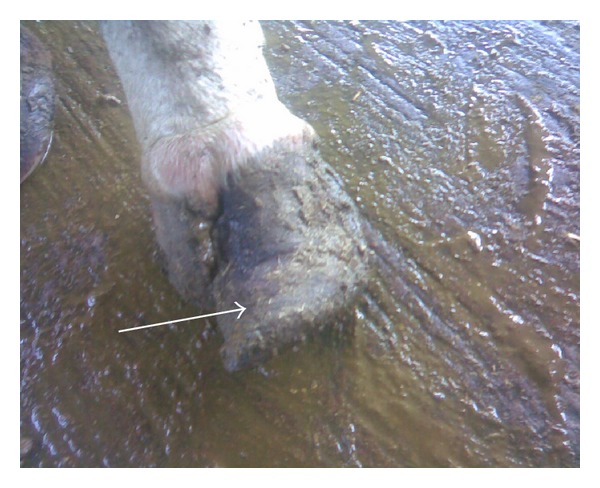
Overgrown hoof.

**Figure 10:**
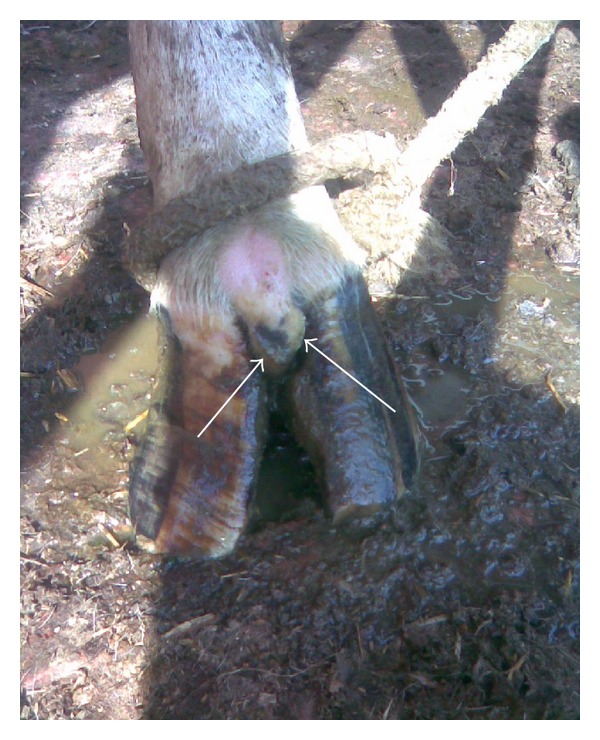
Interdigital hyperplasia.

**Figure 11:**
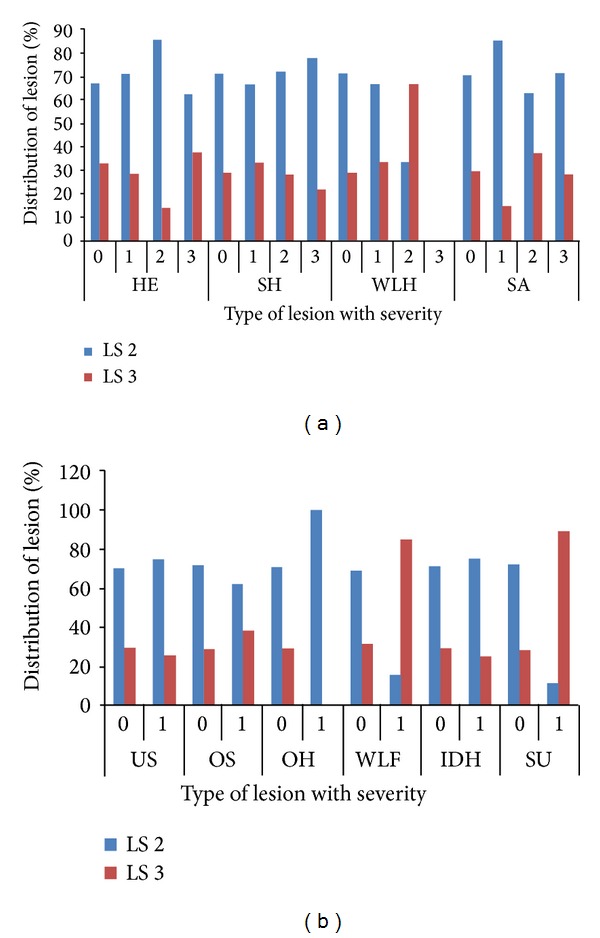
(a) Distribution of severities of lesions in cows with locomotion score 2 and 3, HE: heel erosion, SH: sole haemorrhage, WLH: white line haemorrhage, SA: sole avulsion, LS: locomotion score. (b) Distribution of severities of lesions in cows with locomotion score 2 and 3, US: under run sole, OS: overgrown sole, OH: overgrown hoof, WLF: white line fissure, IDH: interdigital hyperplasia, SU: Sole ulcer, LS: locomotion score.

Out of 450 cows, 8.6% (*n* = 39), 73.7% (*n* = 332), 12.8% (*n* = 58), and 4.6% (*n* = 21) were having 0, 1, 2, and 3 locomotion score, respectively. The association between locomotion score and various lesions is presented in Tables 1 and 2 and the photographs of various lesions and RLVI are depicted in Figures 1, 2, 3, 4, 5, 6, 7, 8, 9, and 10. Heel erosions were present in about 70% of the animals with locomotion score 2 and about 28% of the animals with locomotion score 3 (Figures 11(a) and 11(b)) but they failed to cause apparent lameness. In contrast, sole ulcers and white line fissures had a close association with poor locomotion, with figures of 11.11% and 88.89% for sole ulcer and 15.58% and 84.42% for white line fissures, for locomotion scores 2 and 3, respectively (Figure 11(b)). All the other lesions did not cause clinical lameness but tend to increase asymmetry (Table 1).

**Figure 12:**
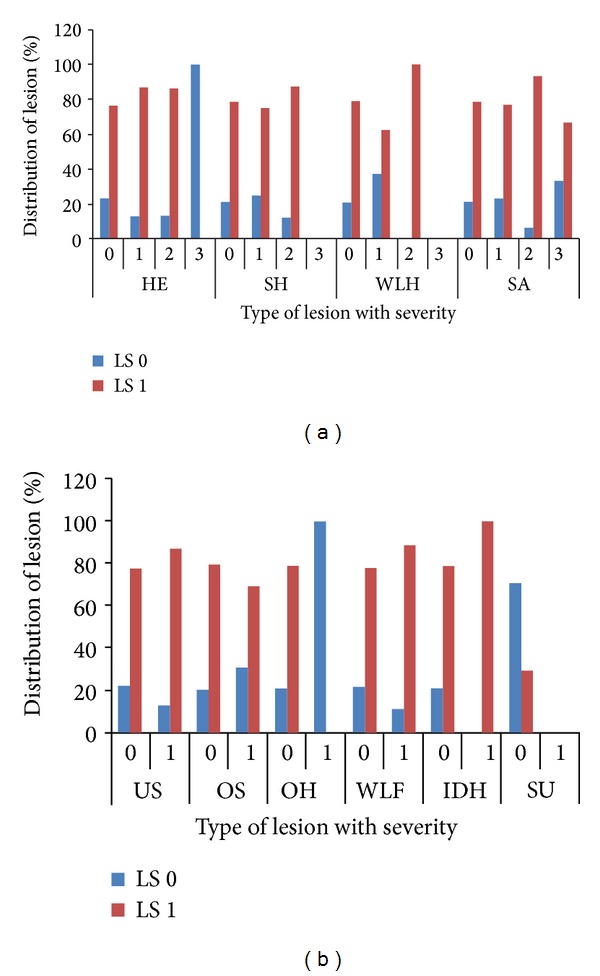
(a) Distribution of severities of lesions in cows with locomotion scores 0 and 1, HE: heel erosion, SH: sole haemorrhage, WLH: white line haemorrhage, SA: sole avulsion, LS: locomotion score. (b) Distribution of severities of lesions in cows with locomotion scores 0 and 1, US: underrun sole, OS: overgrown sole, OH: overgrown hoof, WLF: white line fissure, IDH: interdigital hyperplasia, SU: sole ulcer, LS: locomotion score.

In case of normal animals (locomotion score 0) and asymmetric animals (locomotion score 1) the presence of lesions was associated with increased locomotion score. In other words the degree of asymmetry was more in animals with foot lesions (Figures 12(a) and 12(b)). However, despite the presence of lesions, these animals were not clinically lame.

In clinically lame cows (locomotion scores 2 and 3) there was a significant correlation between sole avulsions and heel erosions (0.26; *P* ≤ 0.05), underrun soles and heel erosions (0.224; *P* ≤ 0.05), overgrown hooves and heel erosions (0.315; *P* ≤ 0.01), overgrown sole and underrun soles (0.299; *P* ≤ 0.01), and sole ulcers and white line fissures (0.253; *P* ≤ 0.01) (Table 3). In cows with locomotion scores 0 and 1 there was a significant correlation between overgrown soles and heel erosions (0.386; *P* ≤ 0.01), white line haemorrhages and sole haemorrhages (0.45; *P* ≤ 0.01), and interdigital hyperplasia and white line fissures (0.884; *P* ≤ 0.01) (Table 4).

There was a significant relationship between locomotion score and RLVI (*χ* ^2^ = 4.87; *P* ≤ 0.05 for locomotion scores 2 and 3; *χ* ^2^ = 12.95; *P* ≤ 0.05 for locomotion scores 0 and 1) to identify lameness. Spearman's rank correlation coefficient for locomotion score from two observers was 0.637 (*P* ≤ 0.01) and that for RLVI was 0.753 (*P* ≤ 0.01).

## 4. Discussion

In the present study, despite the presence of lesions in the animals with locomotion scores 0 and 1, these animals did not show clinical lameness. The reason for this might be that several claw and digital lesions do not seem to inflict sufficient pain to cause clinical lameness [16]. Also, the individual susceptibility to foot lesions, pain, and resulting lameness vary from animal to animal [17]. Furthermore the potentially painful corium insult occurs several weeks before the lesions are visible in the sole [18]. So, ignoring these cows with lesions in the feet may reduce the probability of detecting risks for lesion development by misclassifying these cows as nonlame in spite of having lesions in feet.

From Table 3, it can be inferred that presence of sole avulsions, underrun soles, and overgrown hooves increased the chances of occurrence of heel erosions; presence of overgrown soles increased the chances of underrun soles; and presence of sole ulcers increased the chances of occurrence of white line fissures. The association between underrun sole and heel erosions may be attributed to their common aetiopathogenesis of subclinical laminitis [19]. A disturbance of microvasculature of the corium results in the escape of blood components into the tubules of the horn of the sole and bulb. As recovery occurs and sound horn is produced, the event is recorded as a blood strained stratum. As the new horn grows, the haemorrhagic stratum moves towards the surface, and during wear (or if horn is pared) it appears in the substance of the horn until it is worn away. A haemorrhagic stratum often terminated as a groove on the heel or under running of the sole. This underrunning until avulsed is observed as underrun soles. But if the sole horn quality is poor due to improper nutrition/mineralization the soles may get avulsed leading to sole avulsions [19].

The correlation between overgrown hooves and heel erosions may be attributed to altered balance of the claw with heel erosions leading to more weight bearing on the toes, leading to overgrowth of hooves. The association between underrun soles and overgrown soles could be due to possible damage to the axial solar part of the medial claw initiating the development of underrun sole [19], because most of the overgrown soles were present in the lateral hind claws. The association between sole ulcer and white line fissure may be due to the fact that both appear as a sequel to sole haemorrhages and white line haemorrhages, respectively, which are common lesions associated with subclinical laminitis. Subclinical laminitis causes damage either in lamina portion of white line or in the sole bulb area, the common site of sole ulcer. The significant correlation between sole ulcers and white line fissures and poor locomotion in the present study was in concurrence with that of Tadich et al. [7]. Clarkson et al. [20] also observed that sole ulcer and white line disease were the lesions responsible for lameness while another study [21] demonstrated that the skin lesions, namely, digital dermatitis and interdigital phlegmon, were the important cause of lameness. Similar to previous studies [10, 22] heel erosions and sole haemorrhages were not associated with locomotion score. Correlation between sole ulcer and double sole has been reported previously [7, 23] but no such correlation was observed in our study.

In normal (lameness score 0) and asymmetric animals (lameness score 1), the presence of overgrown soles increased the chances of heel erosions or vice versa and white line haemorrhages increased the chances of occurrence of sole haemorrhages or vice versa. The possible reason for these correlations could be similar to that for the clinically lame animals. White line fissures may have increased the proximity of hooves and interdigital space to the underfoot manure/slurry and may have led to interdigital dermatitis and in turn interdigital hyperplasia [19].

The significant correlation between locomotion score and RLVI indicated that the placement of claws gets disturbed due to pain of different lesions, leading to improper weight bearing. As more lesions were observed in hind lateral claws, the animals shift their weight to medial claws to relieve the pain in lateral claws resulting in inward knuckling of hocks (cow hock rear leg view). This change in the rear leg view conformation along with the type of lesion present may render the animal asymmetric or lame. Thus, a significant correlation between two indices indicates that both locomotion score and RLVI can be reliably used to identify clinical lameness on dairy farms, despite the subjective nature of clinical diagnosis.

## 5. Conclusions

It was concluded that sole ulcers and white line fissures were the lesions responsible for clinical lameness in lactating HF crossbred cows. Other lesions do not cause clinical lameness but tend to increase asymmetry in dairy cows. The presence of sole avulsions, underrun soles, and overgrown hooves increased the chances of occurrence of heel erosions; presence of overgrown soles and white line fissures increased the chances of occurrence of underrun soles and sole ulcers, respectively. Locomotion score can be reliably used to identify clinical lameness in dairy cattle.

## Figures and Tables

**Table 1:** Association between locomotion scores (2 and 3) and various lesions.

Lesions	Coefficients	Standard error	*P* value	Lower 95%	Upper 95%
Intercept	2.107	0.116	0.00	1.876	2.339
HE	−0.0029	0.014	0.842	−0.031	0.026
SH	0.0027	0.027	0.921	−0.051	0.056
WLH	0.1100	0.11	0.322	−0.11	0.329
SA	0.0117	0.014	0.392	−0.015	0.039
US	−0.0047	0.049	0.925	−0.103	0.094
OS	0.0391	0.071	0.586	−0.104	0.182
OH	−0.0465	0.226	0.838	−0.499	0.406
WLF	0.0707∗	0.034	0.044	0.002	0.14
IDH	0.0680	0.224	0.762	−0.38	0.516
SU	0.478∗	0.147	0.002	0.184	0.773

*Significant at *P* ≤ 0.05.

HE: heel erosion, SH: sole haemorrhage, WLH: white line haemorrhage, SA: sole avulsion, US: underrun sole, OS: overgrown sole, OH: overgrown hoof, WLF: white line fissure, IDH: interdigital hyperplasia, SU: sole ulcer.

**Table 2:** Association between locomotion scores (0 and 1) and various lesions.

Lesions	Coefficients	Standard error	*P* value	Lower 95%	Upper 95%
Intercept	0.71	0.088	0.000	0.537	0.890
HE	0.015	0.013	0.263	−0.012	0.042
SH	0.010	0.035	0.780	−0.060	0.080
WLH	0.006	0.093	0.948	−0.180	0.192
SA	0.003	0.026	0.894	−0.049	0.055
US	0.068	0.044	0.128	−0.020	0.157
OS	−0.146	0.086	0.095	−0.317	0.026
OH	−0.741	0.427	0.088	−1.597	0.115
WLF	0.039	0.143	0.784	−0.247	0.325
IDH	0.021	0.095	0.824	−0.169	0.211

HE: heel erosion, SH: sole haemorrhage, WLH: white line haemorrhage, SA: sole avulsion, US: underrun sole, OS: overgrown sole, OH: overgrown hoof, WLF: white line fissure, IDH: interdigital hyperplasia.

**Table 3:** Correlation between lesions within cows having locomotion scores 2 and 3 (first row = correlation coefficient, second row = probability value).

Lesion	HE	SH	WLH	SA	US	OS	OH	WLF	IDH	SU
HE	1									
	*·*									

SH	0.1	1								
	0.201	*·*								

WLH	−0.019	−0.047	1							
	0.332	0.346	*·*							

SA	0.26∗	0.008	−0.07	1						
	0.013	0.473	0.277	*·*						

US	0.224∗	0.003	−0.025	−0.083	1					
	0.029	0.49	0.415	0.243	*·*					

OS	−0.051	−0.163	0.125	−0.083	0.299∗∗	1				
	0.333	0.084	0.145	0.244	0.005	*·*				

OH	0.315∗∗	0.025	−0.041	−0.011	0.096	−0.062	1			
	0.003	0.415	0.364	0.462	0.211	0.301	*·*			

WLF	−0.02	−0.076	0.082	0.123	0.029	0.134	−0.081	1		
	0.433	0.26	0.244	0.149	0.404	0.128	0.249	*·*		

IDH	−0.121	0.084	−0.084	−0.053	0.089	0.033	−0.028	−0.087	1	
	0.154	0.241	0.239	0.327	0.227	0.391	0.406	0.233	*·*	

SU	−0.059	−0.104	0.041	−0.181	−0.189	−0.028	−0.039	0.253∗∗	−0.081	1
	0.31	0.191	0.364	0.062	0.055	0.406	0.37	0.015	0.249	*·*

*Significant at 0.05 level; ∗∗significant at 0.01 level.

HE: heel erosion, SH: sole haemorrhage, WLH: white line haemorrhage, SA: sole avulsion, US: underrun sole, OS: overgrown sole, OH: overgrown hoof, WLF: white line fissure, IDH: interdigital hyperplasia, SU: sole ulcer.

**Table 4:** Correlation between lesions within cows having locomotion scores 0 and 1 (first row = correlation coefficient, second row = probability value).

Lesion	HE	SH	WLH	SA	US	OS	OH	WLF	IDH
HE	1								
	*·*								

SH	0.151	1							
	0.225	*·*							

WLH	0.025	0.45∗∗	1						
	0.842	0	*·*						

SA	0.093	0.241	0.17	1					
	0.456	0.051	0.173	*·*					

US	−0.062	−0.107	−0.141	−0.096	1				
	0.623	0.392	0.26	0.445	*·*				

OS	0.386∗∗	−0.038	−0.081	−0.016	0.205	1			
	0.001	0.764	0.516	0.899	0.099	*·*			

OH	−0.053	0.08	0.162	−0.039	0.008	0.103	1		
	0.672	0.522	0.193	0.759	0.951	0.412	*·*		

WLF	0.109	0.011	−0.114	0.097	0.062	0.101	0.095	1	
	0.384	0.929	0.364	0.438	0.622	0.422	0.45	*·*	

IDH	0.034	0.014	−0.119	0.146	0.143	0.006	0.044	0.884∗∗	1
	0.785	0.91	0.343	0.242	0.254	0.964	0.728	0	*·*

**Significant at 0.01 level.

HE: heel erosion, SH: sole haemorrhage, WLH: white line haemorrhage, SA: sole avulsion, US: underrun sole, OS: overgrown sole, OH: overgrown hoof, WLF: white line fissure, IDH: interdigital hyperplasia.
